# Erosion of traditional ecological knowledge under conditions of hydrosocial rupture: Insights from the Mekong floodplains communities

**DOI:** 10.1007/s13280-025-02172-2

**Published:** 2025-04-24

**Authors:** Thong Anh Tran, Jonathan Rigg, Van Huynh Thanh Pham, Ming Li Yong, Oc Van Vo, Phu Xuan Pham, Phu Thanh Dang, Hieu Van Tran, Cam Hong Thi Nguyen, Thanh Duy Vo

**Affiliations:** 1 Fenner School of Environment and Society, College of Systems and Society, The Australian National University, Canberra, ACT Australia; 2 Faculty of Agriculture and Natural Resources, An Giang University, Vietnam National University Ho Chi Minh City (VNU-HCM), Long Xuyen City, An Giang, Vietnam; 3 School of Geographical Sciences, University of Bristol, Bristol, UK; 4 Faculty of Agriculture and Natural Resources, An Giang University, Vietnam National University Ho Chi Minh City (VNU-HCM), Long Xuyen City, An Giang Vietnam; 5 Research Program, East-West Centre, Honolulu, HI USA; 6 Climate Change Institute, An Giang University, Vietnam National University Ho Chi Minh City (VNU-HCM), Long Xuyen City, An Giang Vietnam

**Keywords:** Climate change, Hydropower development, Hydrosocial rupture, Seasonal livelihoods, Traditional ecological knowledge, Vietnamese Mekong floodplains

## Abstract

What role does traditional ecological knowledge (TEK) play in resource-based livelihoods under conditions of hydrosocial rupture? Does such knowledge come into its own, or is it sidelined, even eroded and jeopardised? This paper addresses these core questions in the fragile floodscapes of the Vietnamese Mekong floodplains. It examines how TEK is intertwined with transboundary hydrological effects of climate and water infrastructural development, and in situ agriculture-driven development policies. Drawing on qualitative data from in-depth interviews and focus group discussions with relevant stakeholders alongside policy and social media documentations, the paper argues that while TEK is an inherent component of the floodplains communities’ adaptation, its erosion is caused by hydrosocial rupture characterised by compounding climate-development effects and associated floodwater disruptions, agrarian transitions, and rural–urban migration. The paper suggests that TEK should be integrated into adaptive agri-environmental governance policies, allowing floodplains communities to better navigate regional climate-development challenges.

## Introduction

In our present Anthropocene era, the nexus of climate and development has become increasingly complex, demonstrating widespread rupture between nature and society (O’Brien 2021). This rupture, driven by both physical and social processes, is typically characterised by biodiversity loss, environmental pollution, and the degradation of natural resources (Moss 2024), significantly disrupting human relationships with nature. The IPCC (2022) highlights how nature-society rupture has worsened the living conditions of rural populations globally, whose livelihoods rely heavily on natural resources.

Populations in the Lower Mekong Basin (LMB) are faced with hydrosocial rupture, characterised by the severing effects between floods and rural societies as a result of climate change (e.g. reduced rainfall), rampant hydropower development along the Mekong River (Pech and Sunada 2008; Hecht et al. 2019; Hoang et al. 2019), and national governments’ development agendas that prioritise the growth of a commodity-based agricultural economy through water infrastructure expansion (van Staveren et al. 2018; Nguyen et al. 2020). These combined factors have transformed the Mekong floodscapes from the local to transboundary scales (Tran et al. 2024). In the Vietnamese Mekong floodplains, these changing dynamics are prevalent among agrarian communities across various livelihood dimensions. Most notably, they significantly affect the traditional ecological knowledge (TEK) that has been developed and passed down by these communities over generations.

TEK is traditionally grounded in and shapes the ways of life, norms, values, and ‘living with flood’ cultures of Mekong floodplains communities. Historical narratives of adaptation illuminate how the evolution of TEK is deeply intertwined with the adaptability of early Vietnamese migrants when encountering new environments in the delta, dating back to the ‘March to the South’ in the mid-eighteenth century (Biggs 2010). Their pioneering spirit and determination to explore these new lands enabled them to learn, innovate, and adapt their livelihoods to the natural environments that confronted them (Taylor 2001; Liao et al. 2016). While TEK plays a crucial role in nurturing the floodplains communities’ lifeworlds and livelihood strategies, there is a lack of understanding of how it is jeopardised by the compounding climate and development challenges at the regional and delta scales.

The complex climate and development dynamics have drastically altered interactions and relationships between Mekong floodplain communities and their environments. Flood disruptions, coupled with environmental degradation driven by large-scale investment in water infrastructure for multi-crop systems and the overuse of agrochemicals, for instance, have led to a significant depletion of natural fish stocks and flood-based resources which provide a vital source of food and seasonal income for millions of rural inhabitants, particularly the poor (Tran and Tortajada 2022; Tran et al. 2023). Additionally, agrarian transitions together with regional and local political economy dynamics have triggered a surge in rural–urban migration, posing challenges to the preservation and transmission of TEK to younger generations in rural areas. Although numerous studies have addressed these issues across geographical localities of the Vietnamese Mekong Delta (VMD) (Geest et al. 2014; Tran 2019; Bayrak et al. 2023; Tran and Touch 2024), the ways in which changing hydrosocial relations are driving TEK erosion remain relatively under-explored.

This paper addresses this knowledge gap by exploring how Mekong floodplains communities have experienced TEK erosion while adapting to new water challenges. Contextualised in the ongoing transboundary and in situ climate and development-driven perturbations in the Mekong floodplains, this paper investigates how hydrosocial rupture is associated with TEK erosion, and how this has impacted agrarian communities’ livelihoods. Drawing on qualitative data gathered from interviews and focus group discussions with respondents across three study areas in the floodplains (An Giang, Dong Thap, and Can Tho), and analyses of policy and social media documents, the paper argues that, while TEK remains an integral part of the everyday livelihoods of the floodplains communities, it is being adversely affected by hydrosocial rupture characterised concomitantly by natural flood disruptions, agrarian transitions, and rural–urban migration.

The paper contributes to the understanding of how TEK is jeopardised by changing hydrosocial relations resulting from the Mekong’s climate and development complexities. From a policy perspective, it highlights the need to preserve TEK through agrarian communities’ everyday livelihood practices as well as integrate such knowledge into adaptive agri-environmental policies on a broader scale. Such integrative strategies will help foster more effective adaptation pathways for agrarian communities to better tackle climate-development challenges.

## Conceptual framework

### Hydrosocial rupture

The Anthropocene era reveals increasing evidence of socio-ecological disruptions resulting from the impacts of human activities on the Earth system (Hamilton 2016), reflected in a rupturing of the relationships between humans and nature (UNEP 2021). Here, the term ‘rupture’ reflects the significant alterations in hydrosocial relationships within the predominantly human-dominated environment. In other words, it refers to the state of existing relationships being broken or disturbed. Mahanty et al. (2023) identify four key dimensions of rupture: the synergistic spatial, multi-scalar, and material drivers of rupture; temporal dynamics of change or crisis; heightened uncertainty, insecurity, and material deprivation; and generative potential of rupture. When applied to the Mekong’s complex hydrosocial dynamics, rupture can be seen occurring across spatial boundaries with long-term cascading effects (Miller et al. 2021; Mahanty et al. 2023). While rupture embraces disruptive effects, it also provides opportunities for fostering positive change (Cretney and Nissen 2023).

River deltas are seen as hotspots of hydrosocial rupture (Hoitink et al. 2020). The concept is used to “denote moments of profound change in human-water relations that traverse administrative boundaries and exceed the governance capacities of jurisdictions, sectors, and individual groups of resource users” (Miller et al. 2021, p. 20). As such, the concept resonates with the Mekong’s complex climate-development context. In the lower Mekong countries, for example, these complexities have progressively strained efforts to secure biodiversity, water, food, and energy for riparian populations. Hydropower development, among other development interventions, causes a “dramatic disruption of land, water, and social relations” (Chann et al. 2024, p. 316). These also demonstrate sustained efforts of the Mekong floodplains communities in (re)innovating their livelihood approaches to better accommodate change. Such efforts extend beyond individual actions to include the social, cultural, and institutional dimensions of hydrosocial relationships (Beery et al. 2023).

Understanding hydrosocial rupture in the Mekong floodplains needs to be grounded in the multi-scalar, transboundary, in situ, and temporal effects of climate change, hydropower development, and the local economy-driven development agenda. By examining hydrosocial rupture in relation to the simultaneous effects of flood disruptions, agrarian transitions, and rural–urban migration across the case study areas, we aim to understand how these compounding factors have shaped the livelihoods of agrarian communities, and how the resulting changes in hydrosocial relations account for the evolution and the diminishing effects of TEK over time. Given the manner in which hydrosocial rupture threatens the livelihoods of floodplains communities (Miller et al. 2021; Mahanty et al. 2023), TEK becomes a potential means to tackle climate and development-driven challenges. This then raises the question of how TEK can be successfully integrated with scientific knowledge to better support rural livelihoods on the wider community scale.

### Conceptualisation of TEK

TEK is defined as “a cumulative body of knowledge, practice, and belief, evolving by adaptive processes and handed down through generations by cultural transmission, about the relationship of living beings (including humans) with one another and with their environment” (Berkes et al. 2000, p. 1252). It can be identified through various terms that are often used interchangeably, including indigenous and traditional knowledge (ITK), indigenous knowledge (IK), local knowledge (LK), and indigenous and local knowledge (ILK). Regarded as ‘native science’ (Cajete 2018), TEK is developed through the co-evolving processes of human experimentation, learning, and interactions with environments (Tengö et al. 2014). Geographically, it is bounded to a smaller spatial and longer temporal scale, and to more specific localities than relational scientific knowledge (Gagnon and Berteaux 2009).

TEK has gained prominence and made significant contributions to various research areas, including climate change adaptation, natural resource management, biodiversity conservation, and resilience (Ford et al. 2020; Mekonnen et al. 2021; Rai and Mishra 2022; Peter et al. 2024). Much has been learned about how communities in the Global South utilise TEK to address disruptive socio-ecological systems and other external challenges (Baird et al. 2021; Tagliari et al. 2023; Poissant et al. 2024). While TEK is embedded in well-established social and cultural practices, beliefs, values, and customs of rural communities (Boafo et al. 2016), it is also expressed through stories, folklore, songs, arts, and other forms of cultural expression (Saxena and Rao 2018).

TEK has encountered widespread erosion. Some studies attribute this to climate change (Pearson et al. 2023; Boafo and Yeboah 2024), while others highlight gaps in the transfer of experience and knowledge between generations (Okui et al. 2021). Social and political dynamics also contribute to the decline in TEK transmission, including formal education, migration, and political movements of peasants (Gruberg et al. 2022).

It is essential to highlight how TEK evolves under the effects of hydrosocial rupture. TEK is not static but continuously adapts to changing environments and technologies (Baird and Manorom 2019). Framed as ‘a process’, the evolution of TEK involves a successive process of ‘observing, learning, and adapting’ (Berkes 2012). By exploring hydrosocial rupture through vignettes of agrarian communities’ life-long engagements with floods in the Mekong floodplains, we illustrate how TEK takes shape, evolves, sustains, and erodes, revealing how such processes make clear the challenges faced by agrarian communities as they seek to adapt to environmental change.

## Materials and methods

### Study areas

**Fig. 1:**
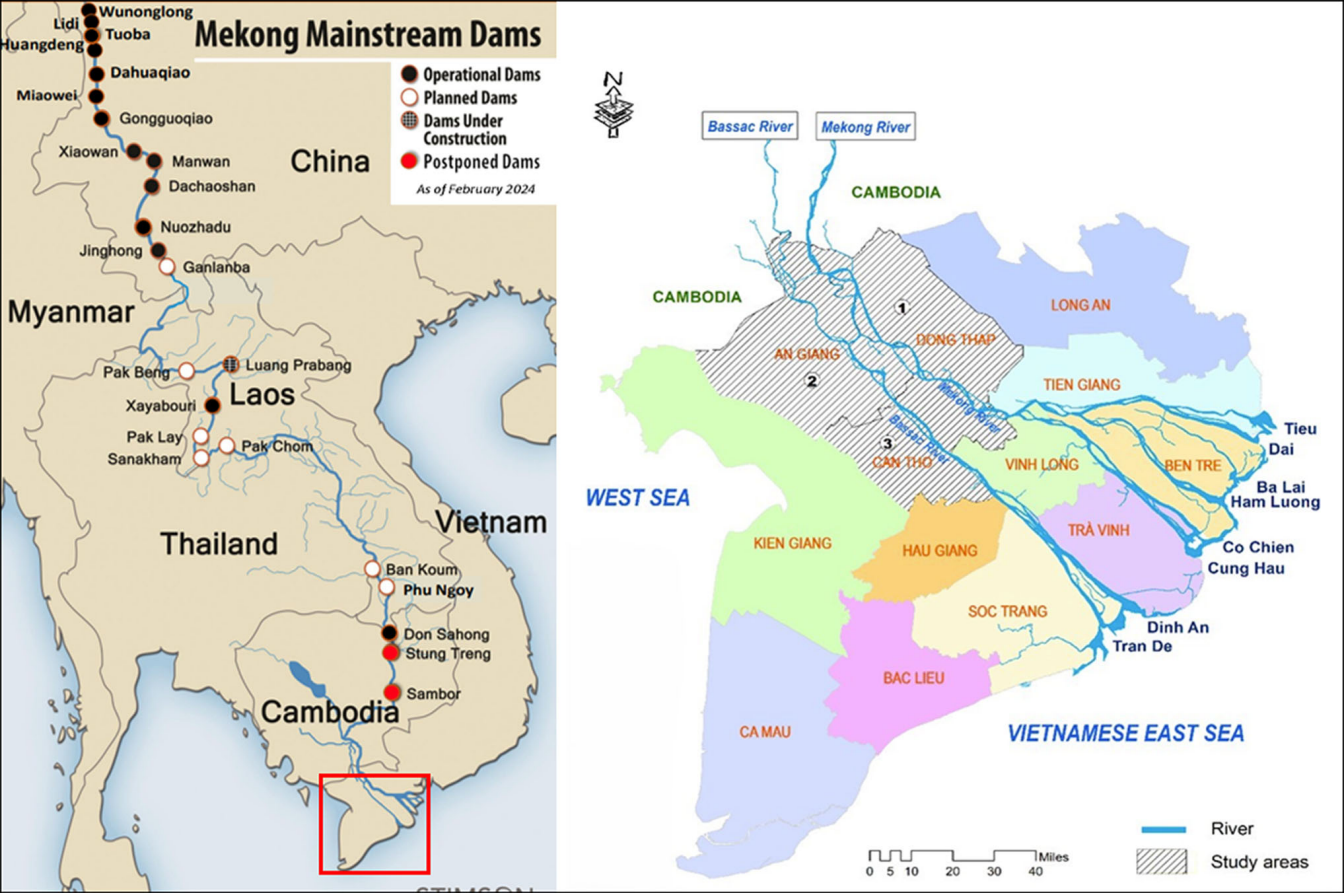
The Mekong region (left) and the study areas (in stripes) in the Vietnamese Mekong floodplains (right) including: (1) Dong Thap, (2) An Giang and (3) Can Tho. Base map source: Eyler and Weatherby (2020)

The study focuses on three provinces in the Vietnamese Mekong floodplains that span the Mekong and Bassac Rivers: (1) Dong Thap, (2) An Giang, and (3) Can Tho City (Fig. 1). Located in the upper part of the floodplains, the first two study areas (Dong Thap and An Giang) experience higher flood levels (≥ 450 cm) (Le et al. 2007). Meanwhile, located in the central part of the floodplains, Can Tho is subject to lower flood depths.

Hydrosocial rupture is particularly evident in the agricultural sector, which serves as the backbone of the VMD’s rural economy. Upstream hydropower operations, coupled with extreme climatic events such as El Niño-induced droughts, and the expansion of water infrastructure (dykes) for intensive agricultural production over the past decades, have led to recurring disruptions of flood flows, drastically affecting agriculture and seasonal livelihood activities in the floodplains (Tran et al. 2024). In the case study areas, community livelihoods are largely shaped by the tropical monsoon climate and upstream water regimes, with the flood season lasting from July to November (Tran et al. 2022). Apart from agriculture, aquaculture, wild fish capture, and various off-farm activities provide the primary sources of income for rural communities.

### Data collection

The paper employed an exploratory qualitative approach to investigate TEK in the context of changing floodscapes in the Vietnamese Mekong floodplains. We combined multiple sources of empirical data collected at different time intervals, including focus group discussions (FGDs) and one-on-one in-depth interviews (INTs) with a diverse range of key respondents from July 2011 to August 2024 (Table 1). In total, these numbered sixteen FGDs and one hundred INTs. These aggregated data enabled the researchers to keep track of (a) alterations in flood patterns over time, (b) their respective impacts on community livelihoods, and (c) their implications for TEK erosion across the study areas. This data collection strategy has been used in numerous empirical studies on climate change adaptation (Simpson et al. 2019; Tran et al. 2022, 2023), aligning well with the objective of the study. Notably, we identified salient legacy, connectivity, and complementarity among the data, which shared common themes, including altered flood regimes, change in community adaptation through flood-based livelihood systems, and the evolution and erosion of TEK over time.

**Table 1:** Data collection methods and key topics investigated across the study areas

Data collection intervals	Data sources	Respondents	Study sites	Topics investigated
Apr to Aug 2024	6 FGDs	Forty-one community members including rice farmers, fish farmers and fishermen	An Phu and Phu Tan, An Giang	Changing floodwater systems (water scarcity) and impacts on traditional farming knowledge systems
	36 INTs	Farmers, agricultural and environmental experts	An Phu and Phu Tan, An Giang	TEK erosion as a result of hydrosocial rupture (change in flood-society) relationships in the Mekong floodplains
			Tam Nong, Dong Thap	
			Co Do, Can Tho	
Sep 2021 to Dec 2022	11 INTs	Farmers, environmental experts, and government officials	An Phu and Phu Tan, An Giang	Changing floodwater systems and impacts on farming activities
				Change in traditional farming knowledge systems
Feb 2019 to Nov 2020	15 INTs	Farmers, agricultural and environmental experts, government officials	An Phu, An Giang and Tam Nong, Dong Thap	Variation in floodwater systems and impacts on aquacultural and agricultural production
Sep 2013 to Mar 2014	9 FGDs	About fifty community members including rice farmers, freshwater prawn/eel farmers, and fishermen	Tam Nong, Dong Thap	Drivers of change in floodwater systems
	33 INTs	Farmers, agricultural and environmental experts, and government officials	Phu Tan, An Giang	Evolution of innovative adaptation (farming systems)
			Co Do, Can Tho	Learning and sharing of traditional knowledge in rice farming and fishing activities
				Farmer and institutional adaptation to changing flood conditions
Jul to Sep 2011	1 FGD	Nine community members involved in fish-trap crafting	An Phu, An Giang	Everyday livelihood practices of rural communities (involved in fish-trap crafting)
	5 INTs	Community members		Impacts of floods on fish-trap crafting and wild fish capture

The data collection targeted respondents who possessed intensive knowledge about or were (in) directly engaged in ‘living with floods’ in the study areas. Specifically, the respondents included government officials, environment and agriculture experts, as well as elderly farmers including rice farmers, fishers, and fish farmers. Across the various data collection intervals we attempted, where possible, to re-interview respondents (farmers and experts) to get a sense of how their views and practices changed over time. This re-engagement process allowed us to communicate with the respondents while capturing emergent insights regarding change in hydrosocial relationships and the relevant issues of TEK erosion in the floodplains.

Most data were collected through face-to-face interactions; however, some interviews were conducted virtually (over Zoom), particularly between November 2020 and September 2021, due to COVID-19 travel restrictions. The questions aimed to explore respondents’ concerns regarding altered flood patterns, their cumulative effects on the depletion of flood-based resources and the disruptions of traditional rural livelihoods, and TEK erosion relevant to these processes. We adhered to research ethics principles through the data collection process. Specifically, before conducting FGDs and INTs, we obtained consent from the respondents, providing an introduction of the research aims and topics and requesting permission to audio-record the sessions. Each FGD/INT lasted approximately one and a half hours.

This paper also involved extensive literature reviews relevant to the research topics, including journal articles, books, scientific reports, and government policy documents. We also collected a wide range of social media content related to climate change, water challenges, and rural adaptation practices in the VMD. This content included personal perspectives from concerned individuals on Facebook, interviews with agricultural and environmental experts, farmers, and relevant stakeholders documented by provincial television agencies on YouTube, as well as articles from national mainstream newspapers. Social media data have been widely utilised in qualitative research (Andreotta et al. 2019; Gangneux 2019) as they provide insights into people’s everyday lives (Sanderson et al. 2024) and help investigate their perceptions and knowledge of the environment (Huang et al. 2013). In this paper, the narratives captured from social media content, combined with empirical insights from the primary data, permits the triangulation of material and offers a comprehensive understanding of hydrosocial rupture and its impacts on the erosion of TEK in the Mekong floodplains.

### Data analysis

Both thematic and narrative approaches were adopted as the primary analytical strategies. This methodology has been widely applied in empirical studies, illustrating how local communities harness TEK in their everyday adaptation strategies in the face of the detrimental effects of climate change (Lejano et al. 2013; Kroik et al. 2020; Guo et al. 2021). Understanding TEK in this sense emphasises the importance of community narratives in storing, communicating, and activating environmental knowledge (Lejano et al. 2013).

In this paper, making sense of hydrosocial relationships derived from combined empirical data and social media analyses helps interpret how TEK evolves and is translated into the everyday adaptation of the Mekong floodplains communities while interacting with changing flood environments. Historical accounts shared by respondents provide a comprehensive understanding of hydrosocial rupture, providing the spatial–temporal interpretations of the contemporary flood regimes and their effects on agrarian communities’ livelihoods, as well as the ways these processes are linked to TEK erosion.

We employed NVivo software for data analysis, applying the grounded theory approach to guide the analytical process (Corbin and Strauss 2015). This approach involves three phases of coding performed including open, axial, and selective coding (Al-Eisawi 2022). Practically, these coding procedures help build the hierarchical patterns of emerging themes while also capturing ‘event stories’ expressed from respondents’ narratives for analysis.

## Results

### Hydrosocial rupture in the Mekong floodplains

Hydrosocial rupture in the Mekong floodplains manifests through the interactive processes between seasonal flood systems and local communities who directly or indirectly engage with flood environments. It demonstrates a radical shift in adaptation away from ‘free adaptation’, in which rural communities live in ‘harmony’ with floods, to ‘forced adaptation’, where they deploy alternative strategies to deal with changing conditions (Tran 2020b). In this regard, the paper highlights a significant decline in natural resources, including floodwaters, which serves as one of the key indicators of hydrosocial rupture.

**Fig. 2:**
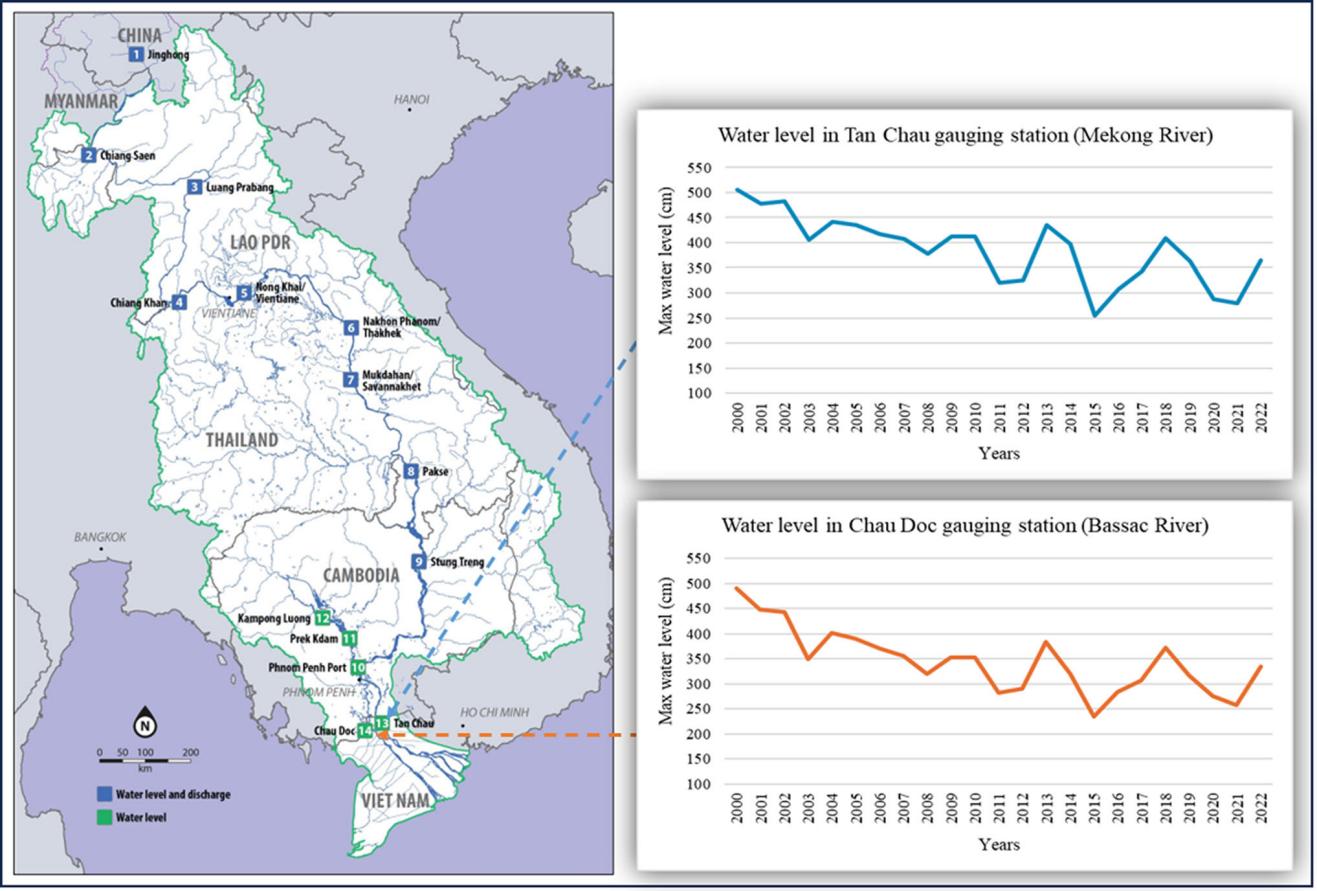
Hydrological stations for flow monitoring on the Mekong mainstream (left) and variations of water levels at Tan Chau and Chau Doc stations in the VMD (right). Sources: LMC Water Centre and MRC (2023) and Statistical Office of An Giang (2023)

Hydrosocial rupture in the Mekong floodplains also manifests at multiple scales. At the regional scale, it is concerned with the compounding effects of climatic events, such as droughts, and upstream hydropower operations which retain a significant amount of water in reservoirs (Hecht et al. 2019). This will be exacerbated by the planned 180-km-long Funan Techo Canal in Cambodia, which is likely to create negative ecological effects for the Vietnamese Mekong floodplains (Yang et al. 2025). These factors contribute to diminishing water flows into the VMD (Fig. 2), affecting the seasonal rhythms of the local water systems and, therefore, agrarian communities’ livelihoods. Reflecting on this hydrosocial rupture, an expert noted:

While erratic rainfalls are commonly observed in the lower Lao areas, water stored in upstream hydropower reservoirs offers additional stress. In the Mekong floodplains [the Long Xuyen Quadrangle], water is not retained in fields but is discharged into rivers and the sea. This environmental chaos is driven by both natural and human factors (INT, September 2024).

Hydrosocial rupture is also attributed to the decade-long agriculture-based development policies implemented in the delta. Agricultural commodification and commercialisation in effect treat the delta as a key resource frontier for rice production aimed at ensuring national security and enhancing rural household income (Chu et al. 2014). In An Giang Province for example, this policy prioritises the adoption of hard infrastructure for flood management, which aims at (1) controlling floodwater infrastructure (or flood control systems), such as North Vam Nao scheme (Tran et al. 2020), and (2) expulsing floodwater out of the delta (Tran and Cook 2024). These technical systems act as the boundary objects that separate humans from floods, perpetuating processes of hydrosocial rupture. As one respondent noted: “I am confident that, in the context of climate change and other complexities, communities that are spatially distanced from nature are getting more vulnerable to their impacts” (INT, September 2024).

### Evolution of TEK: A ‘more-than-human’ process

The evolution of TEK is deeply grounded in the development of social and cultural mechanisms characterised by the values, beliefs, and everyday-life practices of rural communities that guide their adaptation pathways. From our observations, agrarian communities possess profound ontological understandings of natural phenomena which enables them to respond more effectively to changing environmental conditions. TEK enters folklore and proverbs, highlighting how rural farmers ingeniously observe, interpret, and predict varying climate and weather phenomena – such as rain, flood, and drought. This TEK-based capacity has enabled agrarian communities to organise their crop calendars, where they adapt better to nature.

*Chuồn chuồn bay thấp thì mưa, bay cao thì nắng, bay vừa thì râm*

(When a dragonfly is flying low, it is likely to rain

When it is flying high, it is likely to be sunny

When it is flying mid-level, it is likely to be cloudy)

*Trăng quần thì hạn, trăng tán thì mưa*

(A halo around the moon is a sign of drought, diffused moonlight means it is likely to rain)

Narratives of rural adaptation reveal that TEK embodies place-based memories of rural communities, demonstrating reciprocal interactions among individuals with shared interests and common goals, as well as their connectedness to nature. An expert noted that “TEK itself is the outcome of nature-human relationships” (INT, September 2024). As reflected by Duong (2024), the core value of TEK does not aim to prove its accuracy through climate and weather predictions but serves as a glue that links individuals within a rural community and connects humans to nature, integrating nature into their everyday lives. Our analysis suggests a strong sense of rural community connectivity to nature, as portrayed through their engagement with floods for many generations. This is where living TEK is maintained and contributes to (re)shaping the livelihoods of the floodplains communities.

**Fig. 3:**
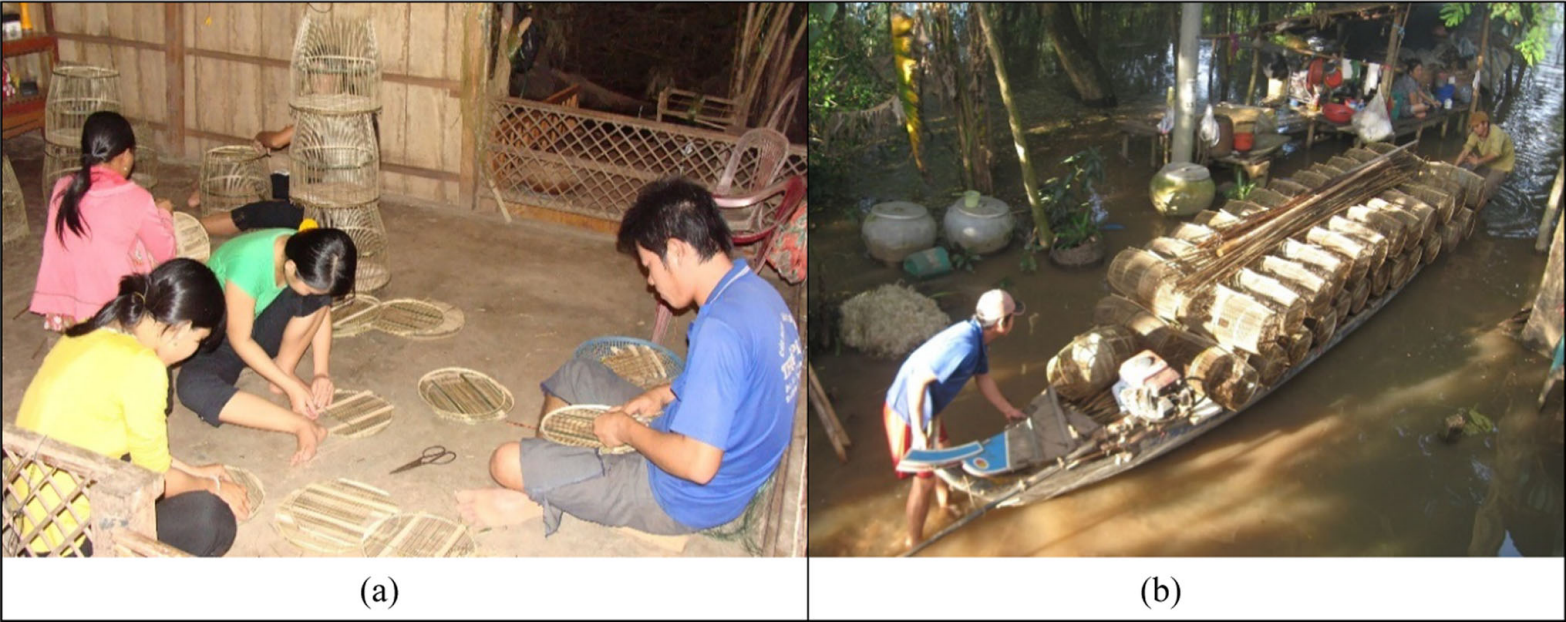
Fish-trap crafting in a floodplain village in An Giang (**a**) and traps used for wild fish capture in the flood season (**b**). Sources: Thanh Duy Vo, 2011

The evolution of TEK is reflected in the manner of the crafting of fish traps and nets, and in other fishing methods by floodplains communities (Fig. 3). Fish-trap makers used to be fishers, possessing enriched experience in ‘fishing for living’ practices. The fish-trap makers cultivated their ecological understandings of flood systems together with fish behaviours and internalised these ‘philosophies’ in the design of the fish traps. Each type of trap is used for a specific fish species, cognisant of habitat conditions. Through trap making, such TEK is taken up and retained within families. It is also shared within bonding networks (e.g. neighbours and friends), who help disseminate this place-based knowledge at a wider community scale. This social learning process contributes to the establishment of ‘epistemic’ fishing communities. Here, TEK can be perceived as a collaborative product of the fishing communities. How it is deployed to support community livelihoods reflects unique socio-cultural aspects of TEK-based adaptation in the Mekong floodplains, where TEK is continuously developed, fostered, and transmitted over generations and over spatio-temporal scales. An expert expressed this as follows:

TEK is socially constructed through regular interactions that extend from family to community members. Social gatherings, such as weddings, create opportunities for knowledge sharing and learning between older and younger generations. These events serve as platforms for discussing crop production and weather-related issues, from which decisions are made. The knowledge generated from these discussions gradually coalesced into TEK, aimed at securing human survival (INT, September 2024).

TEK is also a product of ‘more-than-human’ interactions with nature, highlighting the inherent ethical values and spiritual beliefs held by rural communities concerning natural resource management and environmental conservation. TEK therefore influences rural communities’ behaviours and actions, fostering their connectivity to, and empathy with, nature. Our data suggest that, while the TEK-based ethical values and spiritual beliefs contribute to the protection and preservation of local resources (e.g. fish fingerlings), they also mitigate the use of harmful devices for fishing (e.g. electro-fishing) and promote positive thinking among rural communities for fisheries conservation. One expert explained that:

Using TEK for collecting natural resources enables people to preserve them for future generations. In the past, individuals prioritised catching larger fish over smaller ones. For instance, my mother abstained from buying fish with roe, believing it unethical to kill or eat those kinds of fish (INT, September 2024).

### Erosion of TEK

**Fig. 4:**
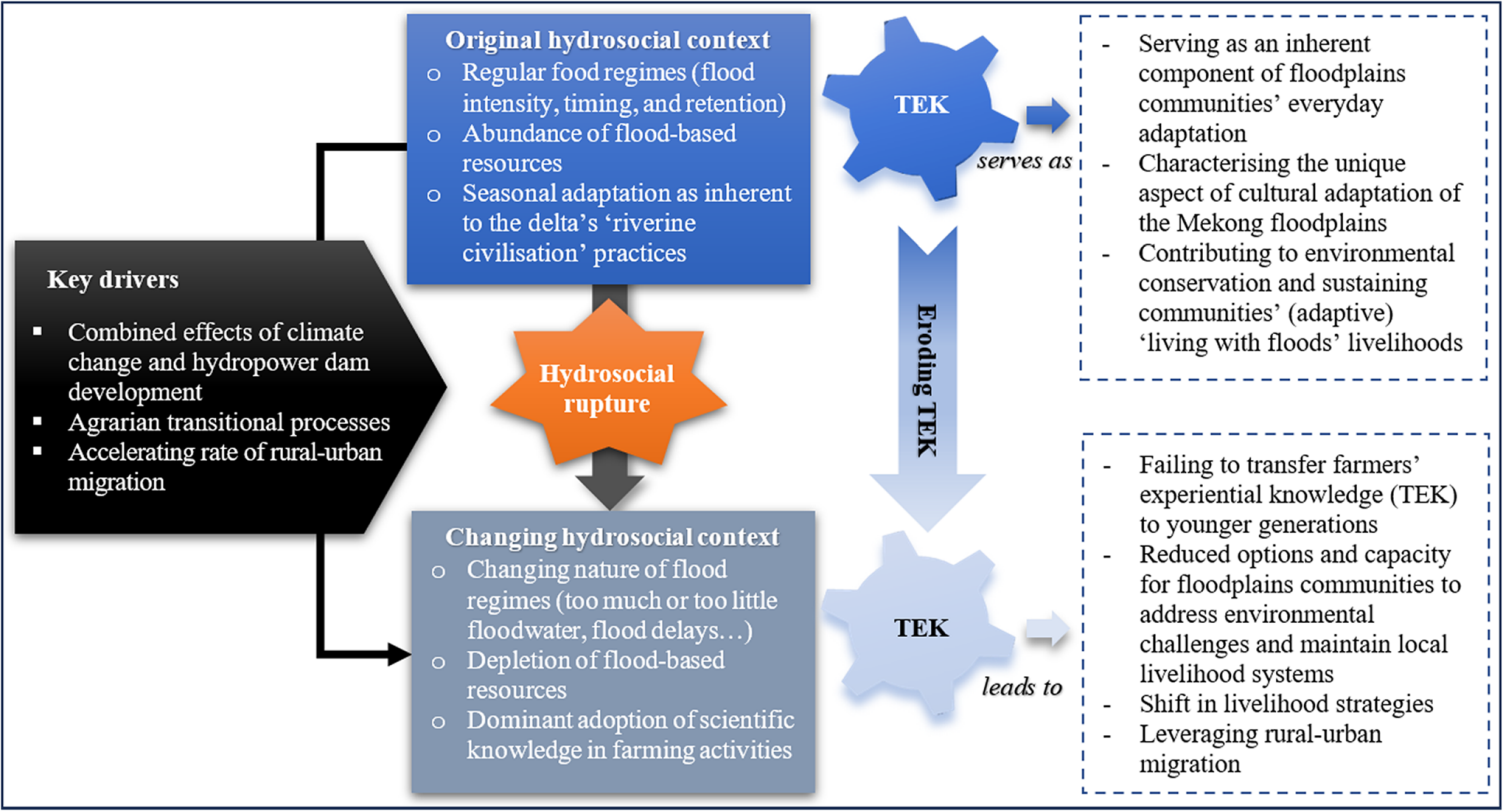
Effects of hydrosocial rupture on TEK erosion in the Mekong floodplains. Source: The first author’s illustrations

Our analysis demonstrates the evidence of TEK erosion taking place across the Mekong floodplains. Three main drivers can be attributed to this, including: (a) climate change and the alterations in the Mekong’s floodscapes, (b) the increasing dominance of scientific knowledge in farming activities due to agrarian transitions, and (c) the breakdown of TEK transmission due to out-migration (Fig. 4). The following section will elaborate on how this has occurred on the ground.

TEK is never fixed in time or place (Table 2). Recent changes in natural flood patterns have led to a community loss of confidence in TEK and its use, evidenced by a sense that their predictions are no longer accurate or that they have lost such knowledge. From our analysis, rice farmers acknowledged that they no longer predict the Mekong’s floodwater patterns accurately. Through their observations in the past, they could make sense of the natural cycles of floods: “*Rằm tháng Bảy nước nhảy khỏi bờ* [Floodwaters flow over riverbanks in the mid-July (lunar month)].” One expert explained that: “TEK can be accurate at one time, but not at another. Given the change in ecological signs of the Mekong floods, it is not always reliable now” (INT, September 2024). The Mekong’s floodwater alterations have led to a ‘rupture’ in communities’ hydrosocial relationships, resulting in a failure to diagnose and predict the physical changes in the floodwater systems. Holding a similar view, another respondent noted that: “In addition to the wider impacts of climate change, transboundary and local human-induced activities have also altered environmental conditions. For example, in An Giang this year [2024], we did not see any signs of flooding in July” (INT, September 2024).

**Table 2:** Summary of drivers and interpretations of TEK erosion in the Mekong floodplains. Sources: Interviews and focus group discussions with respondents

Key drivers	Interpretations of TEK erosion gathered from respondents
Climate change and Mekong’s transformed floodscapes	Environmental conditions are changing rapidly and becoming increasingly unpredictable, making it difficult for TEK to adapt. As a result, TEK has become less accurate and is falling out of use
	The traditional saying “Floodwaters flow over riverbanks in the mid-July (lunar month)” is no longer reliable for predicting floods in the Mekong floodplains
Agrarian transition	TEK has been increasingly alienated by agricultural mechanisation in rural communities. There is growing reliance on scientific knowledge delivered to farmers through agricultural extension agencies and the increased involvement of private sector, such as agrochemical companies, in farming
	Social prejudice regarding traditional farming practices, commonly perceived as obsolete
	There is a decline in the use and discussion of TEK among household members in farming practices, largely due to the introduction of highly efficient and advanced technology. For example, now drones are increasingly used for pesticide spraying (for large farmland areas), replacing traditional methods like hand pumps previously used by farmers
Out-migration	Rural–urban migration disrupts TEK transmission from farmers to their children
	Migrant youth often seek their life away from farming due to its labour-intensive nature and uncertain economic returns
	Gaps in TEK transmission result from diminished interactions or emotionally/spatially broken/weakened ties among family members, especially between migrants (working-age people) and those who remain in rural areas (elderly and children). TEK, therefore, is not passed down across generations

TEK is gradually losing its relevance to the altered hydrosocial relationships in the Mekong floodplains, leading to its erosion in the livelihood operations of fishing communities. Our analysis illuminates a sharp decline in fish-trap crafting villages across the floodplains and the reduced use of fish traps by fishers. This trend can be attributed to two factors. First, the recurring disruptions in the Mekong’s water flows, exacerbated by upstream hydropower dams, have hindered the free migration of fish downstream. Existing mitigation technologies, for example fishways, lifts, and locks, do not inadequately accommodate the large-scale migration of over 50 species (Dugan et al. 2010; Bond et al. 2024). The reduced propagation and retention of waters in fields, canals, or rivers have also diminished suitable habitats for sedentary fish to thrive (Vu et al. 2021). Second, changes in the floodscapes, the ongoing use of agrochemicals in multi-crop rice farming systems (Berg and Nguyen 2018), and the illegal over-exploitation of wild fisheries using electro-fishing devices have collectively depleted fish stocks. The reduced number of fishers engaging in wild fish capture is indicative of a significant decline in fish stocks. One participant in a fish-trap crafting group in An Phu District, An Giang noted: “The diminishing fish-trap crafting is mainly attributed to change in flood levels, the depletion of fish stocks, and the decrease in fish-trap buyers” (FGD, September 2011). A government official of An Phu Office of Agriculture and Rural Development confirmed how this has occurred in recent years: “Previously, there were several fish-trap villages operating in the district [An Phu]. Due to reduced flood flows and the decline in fish stocks, these villages have gradually disappeared. The residents have also shifted to other occupations or migrated to urban areas” (INT, April 2024).

Agrarian transitions in the VMD have induced the alienation of TEK in the agricultural and rural development. At the institutional level, national policies have shifted towards sustainable agriculture and rural development, promoting an ‘agricultural economy’ focussed on transforming traditional ‘agricultural production’ into ‘agricultural economics’ and ‘commodity value chains’ (Prime Minister of Vietnam 2022). At the delta level, this shift has driven the modernisation and industrialisation of agricultural systems to boost agricultural productivity and improve farmers’ economic returns. These processes have resulted in reliance on scientific knowledge on a wider scale, minimising the use of TEK in agricultural practices.

Our analysis revealed several main reasons for the alienation of TEK in agricultural production. First, enhanced agricultural extension services have created favourable conditions for agricultural experts to engage closely with farmers, facilitating the dissemination of scientific knowledge (Tran and Touch 2024). Second, the growing engagement of the private sector, such as agrochemical companies, in providing technical advice and agrochemicals to farmers has further diminished the use of TEK. The expansion of these services, coupled with farmers’ aspirations for higher productivity, has intensified the shift away from traditional (TEK-based) farming practices. Third, there exists a prejudice against traditional farming systems as implied in the term ‘traditional.’ An expert clarified that “As a common sense, ‘traditional’ is equated to outdated or unusable. Traditional farming practices are therefore seen as obsolete.” (INT, September 2024). Fourth, the dominance of advanced technologies in farming, such as the use of drones for pesticide spraying, has sidelined TEK-based farming practices. In this sense, farmers believed that hand pumping (for pesticide application) was comparatively more time-consuming and labour-intensive. As one respondent observed: “Farmers are constantly seeking new farming methods to reduce labour. The increased use of machinery in farming has replaced manual work, gradually leaving TEK out of farming” (INT, September 2024).

Loss of knowledge is linked to loss of knowledge transmission (Okui et al. 2021). In this study, the transmission of TEK is challenged by the surge of rural–urban migration. Rather than farming, many younger generations in the floodplains opt for out-migration to seek alternative income-earning opportunities as a preferred livelihood strategy (Geest et al. 2014; Tran 2019; Tran and Touch 2024). This shift in employment creates a generational gap in TEK transmission, where the knowledge accumulated by grandparents or parents cannot be passed down to their children, putting TEK at high risk of being eroded or even lost over time. Rural youth have also lost their interest in farming, as it entails physically demanding labour under harsh weather conditions but yields precarious economic returns. One respondent noted that: “Out-migration contributes to eroding TEK, as migrants have left their rural homes and the ecosystems they engaged with” (INT, September 2024). The evidence of TEK erosion due to youth out-migration raises a pressing question concerning who will farm in the future (Toumbourou et al. 2023).

There was a ‘sense of regret’ concerning TEK erosion among the respondents. For instance, a prawn farmer in a flood-prone village of Tam Nong District expressed that: “I feel so regretful about the discontinuation of the giant prawn farming that I initiated in this area. It brought profitable returns, but flood alterations have not made it viable any longer. People have to shift to other occupations” (INT, September 2024). Some fish farmers expressed their regrets about fish losses caused by water pollution and the declining trend of this aquacultural sector in the area. A fish farmer in An Phu, for instance, noted: “The change in water quality [water pollution] has serious impacts on my fish. I have used the traditional knowledge acquired from my fellows in resolving this, but there are lots of risks and uncertainty… So far, there has been a significant reduction in cage fishing in this area.” (INT, April 2024). Our analysis, however, suggests that such regrets emerging through hydrosocial rupture may also create an evolving opportunity for farmers to replace obsolete or non-usable knowledge with more innovative and adaptable practices, which are more realistically applicable according to changing hydrosocial circumstances. This implies that rural communities are not passive in the face of environmental changes but actively adapt their TEK to better respond to evolving conditions. As one expert reflected:

“In essence, TEK needs to be regularly discussed among community members. This allows them to update and retain workable knowledge, while discarding the outdated one. Humans are naturally inclined to develop new approaches for survival. The evolution of TEK should be an active part of this process” (INT, September 2024).

### Integrating TEK in adaptive agri-environmental policies

TEK has a clear place in the achievement of climate-resilient and sustainable adaptation strategies in the VMD, where the ‘living TEK’ can be integrated into agri-environmental policies. At the national level, the Vietnamese government has emphasised the importance of incorporating TEK into the sustainable transformation of the national food system by 2030 (see item 2.4) (Prime Minister of Vietnam 2023), as well as their commitment to achieving the 2030 Agenda for Sustainable Development (see objective 2.5) (Prime Minister of Vietnam 2017). At the local level, evidence illustrates keen efforts by governments and agrarian communities in the floodplains in leveraging TEK and incorporating it into technical knowledge for the successful implementation of adaptive agri-environmental policies. Linking this to rice farming, an expert noted: “Cultivating traditional rice varieties in floodplains areas contributes not only to conserving local ecosystems, such as fish stocks, but also to sustaining the livelihoods of the floodplains communities” (INT, September 2024).

The promotion of such adaptive agri-environmental policies highlights the need to integrate TEK into the national government’s climate-resilient and sustainable development strategy, known as the ‘*thuận thiên*’ (living with nature) approach, widely implemented in the delta (Vietnamese Government 2017). The philosophy behind this approach is essentially rooted in the rural community-held TEK, where humans continuously learn to adapt to environments through ‘harnessing nature to deal with nature.’ One expert noted: “Without TEK, we can hardly implement the ‘living with nature’ approach. Based on our understandings of the changing nature of the Mekong water environments, we can manoeuvre our ways to live with it successfully” (INT, September 2024).

Despite its widespread erosion, our analysis suggests that TEK can be preserved in several ways. First, agrarian communities can maintain TEK through their everyday ‘living with nature’ approach. For example, maintaining ecological rice farming systems (e.g. floating rice) in An Giang helps preserve this traditional farming practice among agrarian communities. Second, TEK can be preserved through the collective memory of community members. For example, casual gatherings within family members (e.g. family events) or among rural communities, thus allowing them to reflect how TEK contributes to fostering traditional livelihoods and empowering communities to respond to environmental challenges. One expert noted: “When rural people are drinking or singing folksongs, they are able to remember TEK and share it widely among community members” (INT, September 2024). Third, it is equally important to bring youth back to rural areas, where they can interact with local people, especially with the elderly, acquire TEK and practise it in collaboration with community members.

## Discussion

### TEK erosion and the logics of substitution

Rural communities rely on TEK for innovation and adaptation (Gruberg et al. 2022). In this context, they are not ‘agentless’ or helpless but in a position to leverage their innovative capacity and localised knowledge to navigate environmental complexities (Ford et al. 2020). Our case studies reveal that floodplains communities have enhanced their TEK-based capacity through engagement in ‘learning-to-adapt’ practices (Tran 2020a). This process enables them to accumulate, select, and transmit TEK across agrarian communities and between generations. The finding resonates with the case of nomadic herding in Mongolia, where TEK is passed down inter-generationally among herders as they respond to changes in pastoralism (Peter et al. 2024). Together, these insights reinforce the view that TEK is “an attribute of societies with historical continuity in resource use practice” (Berkes et al. 2000, p. 1252).

TEK erosion is linked to a gradual decline in its transmission over time (Okui et al. 2021). This paper demonstrates that hydrosocial rupture resulting from the interlocking effects of flood disruptions, agrarian transitions, and out-migration have undermined the ‘agency’ and usefulness of TEK, which is essential for livelihood resilience. Pilgrim et al. (2008) found that this phenomenon occurs not only in high-income countries but also in low- and middle-income counterparts, where economic growth policies have increasingly separated humans from their environments. This paper elucidates how this trend will continue to have ripple effects on hydrosocial relationships in the Mekong floodplains, severing inter-generational connectivity in TEK transmission.

**Fig. 5:**
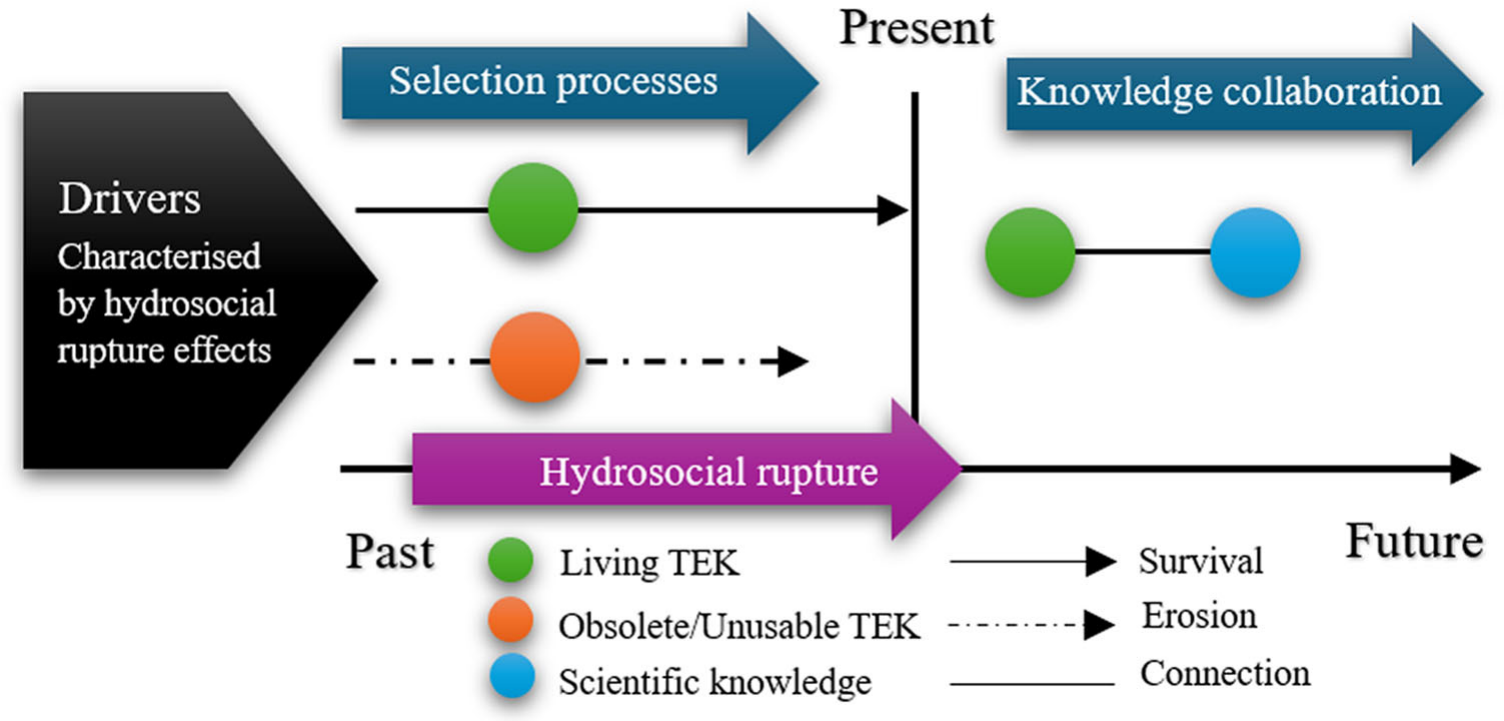
TEK evolution in the Mekong floodplains. Source: The first author’s illustrations

Analysing the breakdown of TEK transmission reveals the practical aspect of rural communities’ adaptation through knowledge selection. Some knowledge remains relevant only for a limited period due to changing environmental conditions. In this vein, knowledge that becomes obsolete or ‘not-fit-for-use’ will be removed from the existing TEK systems and replaced with more innovative and adaptable alternatives that better support communities’ adaptation (Fig. 5). This challenges Kimmerer’s (2018) perspective that TEK erosion signifies rural communities’ inability to adapt due to resource shortages or ecological and economic shifts. In our study, the erosion of the knowledge which has lost relevance paves the way for the development of more innovative and adaptable knowledge, which is pragmatically crucial for community adaptation. In line with Wantzen (2024), this paper argues that addressing hydrosocial rupture does not mean reverting to the past but rather creating new or revising TEK by a collective effort to learn from nature, embrace evolving values in natural resource management, and foster innovation. That said, where ruptures are too deep or too sudden, TEK’s ability to adapt and modify may be compromised.

### Engaging TEK in adaptive agri-environmental governance

While hydrosocial rupture disturbs traditional relationships between humans and nature, it can also catalyse the revitalisation of TEK systems. This echoes a villager-led study in Thailand that aimed to systematically document TEK and use it to reinforce senses of identity and expertise among villagers, and to counter large-scale development projects along the Salween River, and the Mekong River and its tributaries (Lamb 2018; Yong 2020). In the context of the VMD, TEK serves as a vital asset of rural communities and therefore should be preserved and transmitted to younger generations. This is crucial for maintaining traditional adaptation strategies and behaviours of floodplains communities, first developed by early migrants to the delta (Taylor 2001).

Integrating TEK into adaptive agri-environmental governance in the Mekong floodplains is crucial for establishing pathways towards livelihood sustainability and environmental conservation. While there is a ‘rule of selection’ that eliminates obsolete knowledge from the TEK systems, it is equally important to integrate innovative and adaptive TEK into scientific knowledge frameworks. This integration fosters knowledge consilience, addressing perceived shortcomings of each knowledge strand while also enhancing their applicability. As Biedenweg et al. (2023) pointed out, issues affecting humanity cannot be solved without integrating knowledge. This perspective aligns with Riedlinger’s (1999, p. 432) assertion that: “neither Western science nor traditional knowledge is sufficient in isolation for understanding the complexities of global climate change and its manifestations at the local or regional scale”. In the context of the Mekong floodplains, this paper resonates with such claims by arguing that the national government’s climate-resilient and sustainable development strategies (Resolution 120) can be effectively implemented if TEK is recognised and meaningfully integrated into adaptive agri-environmental governance at the farm level and in the wider development context of the Mekong floodplains.

It is critically important to highlight the role of TEK custodians, whose livelihoods are closely intertwined with, and directly affected by, hydrosocial rupture. Our study identifies some key actors who play a key role in this regard, including rural community members such as rice farmers, fishers, and fish farmers, who are the main holders and contributors of TEK. In collaboration with government officials, other stakeholders, including agricultural and environmental experts, they play a crucial role in facilitating the integration of TEK into adaptive agri-environmental governance. This collaboration serves as an essential mechanism and builds a momentum for creating and preserving TEK in the longer term. However, the paper raises an important question: To what extent can this approach warrant the equitable inclusion of TEK, given the dominant role of scientific knowledge in this domain? We acknowledge that this will not be a straightforward task, given the power dynamics and complexities associated with ‘whose knowledge counts’ (Bergström 2021), even in the realm of TEK, particularly when considered through an intersectional lens.

## Conclusions

The study findings demonstrate the prevailing hydrosocial rupture in the Mekong floodplains, driven by the combined effects of climate change, upstream hydropower development and localised agriculture-based development pursued by local governments. These processes reflect an inevitable transition from ‘free’ to ‘forced’ adaptation and illustrate the evolution and erosion of TEK amidst the concurrent challenges of floodwater disruptions, agrarian transitions, and rural–urban migration. The study contributes to scholarship on environmental stewardship by advancing our empirical understanding of (a) how TEK facilitates the ways floodplains communities have deployed the ‘harnessing the nature to deal with nature’ approach, and (b) how floodplains communities serve as custodians of TEK to navigate these pathways.

TEK illuminates its significance in fostering the rural communities’ livelihoods and the unique socio-cultural aspect of TEK-based adaptation in the floodplains. While there exists a ‘sense of regrets’ among the respondents regarding the erosion of TEK, the paper also highlights an irreversible trend that some obsolete knowledge should be discarded and replaced with more innovative and adaptive alternatives to provide more effective support to community adaptation. It is essential that achieving climate-resilient and sustainable development in the VMD requires integrating TEK into adaptive agri-environmental governance to support the resource-based livelihoods of the floodplains communities. This integrated approach helps address the contestations between intensive agricultural development and environmental conservation, both at present and in the longer term. More broadly, the paper helps advance our understanding of how TEK-based adaptation approaches can help mitigate the adverse impacts of hydrosocial rupture, which are widespread in the LMB and other climate-stressed regions of the Global South.

## Data Availability

The data for this study are available from the corresponding author upon reasonable request.
